# Feasibility, acceptability, and perceived usefulness of a community-evidence-based harm reduction intervention for sexualized stimulant use among Mexican gay, bisexual, and other men who have sex with men

**DOI:** 10.1186/s12954-024-01020-y

**Published:** 2024-05-16

**Authors:** Claudia Rafful, Ricardo Orozco, Daniela Peralta, Leonardo Jiménez-Rivagorza, María Elena Medina-Mora, Nely Gutiérrez, Missael Morales-Gutierrez

**Affiliations:** 1 Faculty of Psychology, Universidad Nacional Autónoma de México, Circuito Ciudad Universitaria 04510, Coyoacan, Mexico City, Mexico; 2 Center for Global Mental Health, National Institute of Psychiatry, Mexico City, Mexico

**Keywords:** Stimulant use, Harm reduction, Community-based interventions, Sexual diversity, Sexual minorities

## Abstract

**Background:**

The use of stimulants and other substances with the purpose of enhancing, maintaining, and prolonging sexual activity is known as sexualized substance use. Also known as chemsex, this pattern of use has been mainly explored in high-income countries. The aim of this article was to assess the feasibility, acceptability, and usefulness of a community- evidence-based harm reduction intervention among Mexican gay, bisexual, and other men who have sex with men (gbMSM) adults who reported sexualized stimulant use in the past 6 months and who were not enrolled in any psychosocial treatment.

**Methods:**

The in-person intervention was designed in partnership with gbMSM who used substances. It consisted of 39 harm reduction strategies before, during, and after episodes of use. The components of the intervention were health and self-care, safety, and psychopharmacology. The intervention was delivered at a university campus, a public recreational space, and an HIV public clinic. Feasibility to deliver the intervention was assessed based on enrolment and completion rates; acceptability through a 28-item, 5-point Likert scale (140 max.) constructed and validated for the Mexican population with good reliability coefficients; usefulness through a 5-point Likert scale (“not useful”-“very useful”) for each of the 39 strategies; and potential behavioral change by subtracting the likelihood of implementing each strategy minus the frequency of use of the technique before the intervention.

**Results:**

Participants (n = 19; recruitment rate = 35.2%; completion rate = 84.2%) rated the intervention as acceptable with a mean score of 121.6 (SD = 7.5). The highest potential for behavioral change was regarding the use of information about the half-life of stimulants, polysubstance use, and overdose prevention.

**Conclusions:**

This intervention is feasible when provided within public health services where potential participants are already in contact. Harm reduction strategies need to surpass sexually transmitted infections prevention and HIV care and focus on substance use and mental health strategies.

## Background

Internationally, amphetamine-type stimulants (ATS) and cocaine are the most commonly used illicit substances after cannabis [1]. Since the early 2000s, Mexico has been one of the main countries manufacturing and distributing ATS in the Americas [2, 3]. Consequently, ATS use in Mexico has also increased; in 2021, 62,678 persons sought treatment for ATS use, 2.7 times more than in 2018 (23,542) [4]. In 2019, ATS became the main substance reported among people seeking treatment, with a higher prevalence than that of alcohol treatment seeking (28.8% *vs*. 26.3%) [5]. In 2022, more than half of substance treatment seeking in public centers was related to stimulant use (46.2% ATS and 6.7% cocaine) [6].

To date, the efficacy of pharmacological treatment on outcomes such as abstinence, cravings, or changes in sexual behavior for cocaine ranges from moderate to low [7]; for ATS is, at most, low [8, 9]. Moreover, psychosocial treatment has not proved to be effective yet [10], with contingency management as the most promising strategy for both cocaine [11] and ATS [12]. To effectively address stimulant use among vulnerable populations, research needs to incorporate the target population’s specific needs [13], which may include treatment outcomes other than abstinence, such as a decrease in risk behaviors [12] and improvement in psychiatric symptoms [14].

Some studies have suggested that most current substance use treatments have not taken into account the intersectionality that gay, bisexual, and other men who have sex with men (gbMSM) may experience [15, 16]. Treatment available has also overseen specific needs related to sexualized substance use (known as chemsex in some settings) [17, 18], which include coping strategies for unpleasant emotions, depressive symptoms, and traumatic events [19, 20]. Furthermore, as with the general population, it has been reported that gbMSM may be more interested in engaging in strategies that go beyond abstinence-based programs [21, 22], such as harm reduction strategies. Although its origins were focused on HIV prevention, currently harm reduction strategies encompass strategies that protect and improve the health of persons who use drugs.

In contexts where harm reduction has not been implemented in public programs, feasibility studies to incorporate these type of strategies have been performed, especially among vulnerable populations such as un-housed persons [23]. Nevertheless, it has been suggested that a wide range of persons who use drugs (PWUD) have not been exposed to evidence-based interventions. This is possibly related to a less visible pattern of substance use than that of vulnerable persons [24] and has remained outside of the scope of public health programs. In Mexico, although harm reduction is mentioned in public discourse [25], it has not yet permeated public health programs. That is, harm reduction strategies in Mexico are provided by community-based organizations, which depend on private international funding sources. These strategies include provision of injecting and smoking paraphernalia, safe-consumption facilities, sexual health care, and mental health referrals, among others.

According to previous studies on sexualized substance use in Latin America, including Mexico, this practice is more frequent among persons with medium and high socioeconomic status, stable income, and access to health services [26–28]. Thus, harm reduction programs and services that are usually targeted to people who have no access to social security are not reaching one of the key populations at risk of substance misuse and health-related consequences. Furthermore, studies about healthcare experiences, mostly performed in high-income countries, highlight the need to tailor the delivery of care to the needs of this population [29, 30].

It is relevant to design psychosocial interventions for stimulant misuse in Mexico that ought to consider the specific needs and input of persons who engage in sexualized substance use. Therefore, this study aimed to design a harm reduction intervention for sexualized stimulant use among gbMSM and to assess its feasibility, acceptability, perceived usefulness, and potential behavioral change.

## Methods

### Sample

**Fig. 1:**
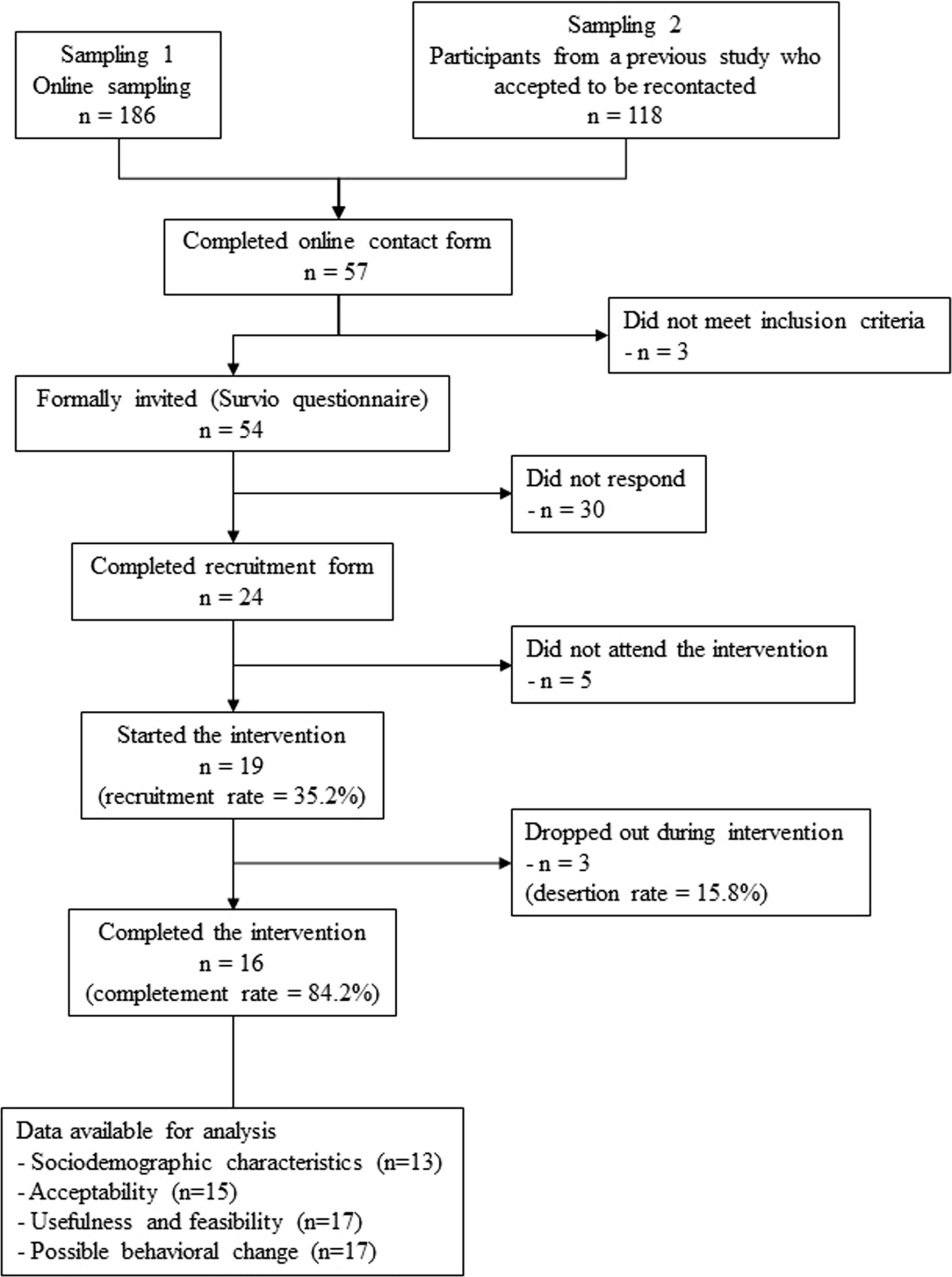
Recruitment and participation flowchart

Participants were recruited through two sampling strategies (Fig. 1). The first one involved an online snowball sampling in collaboration with community-based organizations (CBOs) that provide services to PWUD, and HIV prevention and treatment services in the Metropolitan Area of Mexico City (MAMC). The second sampling strategy consisted of contacting participants of a previous study of this research team, who consented to be contacted for subsequent studies. A full description of the study, that aimed to analyze crystal meth use in the MAMC, is available elsewhere [31]. In both sampling strategies, the inclusion criteria for the present study comprised being 18 years or older, having used stimulants in a sexualized context in the last 6 months, not currently participating in any psychosocial intervention for substance use or mental health, and currently living in the MAMC. Potential participants were invited via e-mail, which included information regarding the procedure, potential risks and benefits, and that their participation would be voluntary and confidential. Following community-based guidelines [32, 33], the invitation to participate also included an online form in which participants reported their availability and schedule preferences. A wide range of options was provided, mainly to determine whether participants preferred a single 4-hour session or shorter sessions, the time (morning or afternoon), and preferred days (weekdays or weekends). All participants opted for a single session on a weekend morning.

### Intervention design/development

For an intervention to be successful, it ought to integrate the needs and concerns of the target group, in this case, gbMSM who engage in sexualized stimulant use. For the development of the intervention, we relied on several sources of information. First, we incorporated the perspectives of the target group based on the results of previous qualitative interviews this study team performed in 2021. Those interviews with 21 participants revealed that most gbMSM initiated crystal meth use in sexualized contexts, by suggestion of sexual partners or based on curiosity after reading comments seen on social media platforms. GbMSM also reported a high treatment need related to their substance use, but that available treatment options lacked a sexual diversity perspective, felt stigmatized by providers, and that such providers were not knowledgeable of the complexity of disentangling substance use from sexual behaviors. Finally, they also revealed a need to better understand how to engage in safer stimulant use practices to prevent negative experiences such as psychotic episodes and overdose.

Second, we incorporated the perspective of 19 health providers who work with persons who use stimulants, especially regarding the practices that are not covered in the public guidelines for substance use treatment [34]. Most health providers reported increased treatment seeking from gbMSM but were not aware of the specific needs of the target population. This implied that they were treating sexualized substance use as any other type of substance use and without a diversity-informed perspective. Moreover, providers that collaborated with harm reduction CBOs reported trying to address this issue considering the complexity of sexualized substance use but without tools or protocols that could guide the potential interventions.

Third, the process was guided by a review of international practices for addressing sexualized substance use among gbMSM [30, 35]. As stated in the introduction, most of the studies have been performed in high-income countries, where the context, while vulnerable and the population stigmatized, differs from the intersectionality that gbMSM in Latin American countries live [36–38]. We incorporated those strategies that were considered acceptable for the context.

### Intervention strategy

The intervention consisted of a brief introduction about harm reduction and three thematic modules (*i.e.*, harm reduction strategies before, during, and after sessions of sexualized stimulant use) with a 10-minute break between them, covering 39 strategies (four in the introduction and 35 divided into the modules). Module 1 (12 strategies) included self-care strategies prior to the substance use session. The strategies covered included healthy eating considering the nutrition requirements persons who use stimulants may have; sleeping hygiene; physical activity; dental, skin, and eye care [because of the high prevalence of nitrate use]; mental health, sexual health; and financial health and safety plans [39–46]. Module 2 (7 strategies) consisted of psychoeducation on the psychopharmacology of stimulants, safety and risks of simultaneous polysubstance use, and self-efficacy while choosing whether or not to continue using drugs in the context of group substance use and sex [47–51]. Module 3 (16 strategies) consisted of strategies to reestablish social activities and health routines after a sexualized substance use session, and participants were encouraged to develop an individual action plan that they could follow in subsequent substance use episodes. The action plan format focused on personal harm reduction strategies for the attendees. The acceptability and perceived usefulness of each strategy was assessed.

The three modules were implemented using active learning techniques, in which participants were encouraged to engage in dynamics. The group facilitators were psychologists who had received constant training in substance use and harm reduction strategies. Examples of active participation included sharing personal experiences, asking questions, and clarifying doubts. The group facilitators promoted a safe space in which participants were encouraged to answer each other’s questions and provide feedback. When a discussion was saturated, the facilitators commented and clarified concepts as needed.

The audiovisual materials for the intervention consisted of a slideshow on the Google Slides platform. Three brochures were made in Canva, a free design and visual communication platform [52]. The brochures aimed to illustrate the content with graphic elements, provided relevant information corresponding to each module, and included Quick Response (QR) codes that redirected to mental health services and other electronic resources of interest.

### Procedure

Between June and August 2023, intervention dates were scheduled in three different locations (*i.e.*, a university campus, a municipal-administered social center, and an HIV public clinic) in Mexico City. These locations were selected to ensure the diversity of participants based on geographic distribution. The university campus is on the South side, the municipal-administered social center is on the East side, and the clinic is on the Central-West side of the city. All locations were easily accessed through public transportation.

Persons who confirmed their assistance received an e-mail reminder of the scheduled date. As the participants arrived at the location, an ID was assigned to ensure confidentiality on each of the forms they were going to complete. Group facilitators provided instructions about the dynamic of each module, asking participants to complete a sociodemographic questionnaire and attendance registration.

In each module, participants received a brochure with the key concepts and QR codes to a web page with the information provided by facilitators. After each module (explained in the Intervention strategy section), participants responded to a questionnaire on the frequency with which they already engaged in each of the strategies presented and their usefulness. After completing the action plan in Module 3, participants answered an acceptability scale, clarified general doubts, and concluded the intervention.

### Measures

Sociodemographic and health characteristics included in the questionnaire were age (continuous), educational attainment (undergraduate and postgraduate degree), employment status (employed, student, public server, homemaking, unemployed), medication intake (open-ended question), and medical conditions (open-ended question).

To assess acceptability, a scale was constructed and validated; this scale has good Bayesian reliability coefficients (Cronbach’s α ranging from 0.757 to 0.968 and McDonald ω ranging from 0.664 to 0.890; every coefficient was calculated for each subdomain) to evaluate seven subdomains: pertinence, effectiveness, loss, gain, convenience, self-efficacy, and affective attitude [Peralta et al., in process]. The full scale consisted of 28 items with a Likert response format, going from 1 (strongly disagree) to 5 (strongly agree). The total score was computed based on the sum of the items, with a maximum of 140 (i.e., high acceptability).

Usefulness was assessed using a scale containing the 39 strategies delivered in the three modules. Each strategy was scored with a Likert response assigned from 0 to 5 according to its usefulness (not useful to very useful). Participants also scored the frequency in which they engaged in each strategy prior to the intervention (0=never- 5=always) and how feasible they considered implementing each strategy.

### Data analysis

Descriptive statistics analyses (frequencies and percentages, and medians and quartiles Q1 and Q3 or means and standard deviations (SD) for normally distributed variables) were conducted for the sociodemographic characteristics, the acceptability scale, and the perceived usefulness questionnaire.

To assess the feasibility of delivering the intervention, we calculated the recruitment rate, the proportion of participants who completed the intervention, and the rate of desertion. We chose these measurements based on previous research in substance use [53, 54]. To assess acceptability, the median for each item of the acceptability scale was obtained, as well as the mean and SD for the total score. Potential behavioral change was computed by subtracting the likelihood of implementing each strategy minus the frequency of its use before the intervention, and significant differences were assessed using the Wilcoxon test. Statistical tests were evaluated at the 0.05 level. All data analyses were performed using R version 4.1.2 in R Studio [55].

### Ethics

All procedures contributing to this work comply with the ethical standards of the relevant national committees on human experimentation and with the Helsinki Declaration of 1975, as revised in 2008. All participants gave informed consent. This study was approved by the Ethics Committee of the Faculty of Psychology, Universidad Nacional Autónoma de México (FPCE_10022022_H_AC).

## Results

### Feasibility to deliver the intervention

Out of the 57 persons who responded to an online form expressing interest in participating (Fig. 1), 54 satisfied the inclusion criteria and were invited to participate and asked to fill out a Survio questionnaire, with 24 completing this recruitment form. In total, 19 participants attended the harm reduction intervention, and 16 completed the 3 modules of the session. The recruitment rate (i.e., the proportion of people who initiated the intervention divided by those formally invited) was 35.2%. The completion rate among those who attended was 84.2%; therefore, 15.8% dropped out during the intervention. Most participants reported having an undergraduate degree (69.2%), being employed (63.6%), and living with HIV and taking antiretroviral therapy (ART) (69.2%) (Table 1).

**Table 1:** Sociodemographic and health characteristics of gay and bisexual men who have sex with men who engage in sexualized substance use in Mexico City (n = 13)

	n (%)
Median age (Q1–Q3) (years)	34 (33–39)
Educational attainment	
Undergraduate studies	9 (69.2)
Postgraduate studies	4 (30.8)
Employment status*	
Employed	7 (63.6)
Student	2 (18.2)
Homemaking	1 (9.1)
Unemployed	1 (9.1)
Medication intake	
ART	8 (61.5)
ART and others	1 (7.7)
PrEP	2 (15.4)
Antidepressants	1 (7.7)
None	1 (7.7)
Medical conditions	
HIV	9 (69.2)
HCV	1 (7.7)
None	3 (23.1)

Q1: quartile 1. Q3: quartile 3. *ART* antiretroviral therapy. *PrEP* pre-exposure prophylaxis

*For employment status n = 11

Participants who dropped out reported having prior commitments scheduled and being unable to stay longer. However, since they did not mention this at the beginning of the intervention, it is possible they were bored or had other reasons to leave.

### Acceptability

Of those who started the intervention, 78.9% of participants (n = 15) completed the acceptability scale (Table 2). For most of the items, participants rated them with the highest score (*e.g.*, Strongly agree). Participants rated lower items related to lifestyle changes or investing time to adhere to the strategies (e.g., My lifestyle will not allow me to maintain the strategies provided in the intervention). Overall, based on the total score mean and standard deviation, participants rated the intervention as very acceptable, with a mean score of 121.6 (SD = 7.5), noting that the maximum score for the scale is 140.

**Table 2:** Acceptability of the intervention (n= 15)

Item	Median (Q1–Q3)
The strategies are clear	5 (5–5)
The intervention is logical	5 (4–5)
The strategies are useful	5 (4.5–5)
It is clear how the intervention can help me	5 (4.5–5)
Good results in the long term	5 (4.5–5)
The intervention may improve my health	5 (5–5)
The intervention could be effective	5 (4–5)
The intervention may negatively affect my health*	5 (5–5)
The intervention may negatively affect my life*	5(4.5–5)
I may lose more than I could gain by participating*	5 (4–5)
It may help me in several aspects of my life	4 (4–5)
Participating will improve my health	4 (4–5)
I may gain more participating	5 (3.5–5)
I may benefit from the strategies learned	5 (4–5)
The strategies may take up too much time*	4 (4–5)
My lifestyle will not allow me to maintain the strategies provided in the intervention*	4 (2.5–5)
It will require too much effort to implement the strategies*	4 (3–4)
I can follow the strategies	4 (4–5)
I can attend the intervention sessions	4 (3.5–5)
I may use the strategies to improve my health	5 (4–5)
I will be able to implement the strategies when needed	4 (4–5)
I can easily complete the intervention	4 (3.5–5)
I am excited to participate	4 (4.5)
I have the motivation to participate	4 (4–5)
I feel an obligation to participate^†,^*	3 (2–4)
I really want to participate	4 (4–5)
I feel bad about participating*	5 (3.5–5)
I feel confident to participate	4 (4–5)

Q1: quartile 1. Q3: quartile 3

^†^For item I feel an obligation to participate n= 14

*Items that were reverse coded due to negative phrasing

### Frequency, usefulness, and likelihood of implementing each strategy

These instruments were completed by 89.4% of participants (n = 17). For each strategy, the median score was calculated for its frequency prior to the intervention, usefulness, and likelihood of implementing each strategy (Table 3). Participants had previous knowledge and used strategies in varying degrees of frequency: low-use strategies included little prior knowledge of the pharmacological characteristics and long-term consequences of stimulants, as well as low frequency of use of self-care practices strategies that could be implemented during sexualized substance use sessions, that included the health economy strategies (e.g., blocking cards and bank apps). High-use strategies included HIV testing, overall medical care, and physical safeguards.

**Table 3:** Usefulness and feasibility of the harm reduction intervention for each module (n= 17)

	Frequency of use of the strategy before the intervention	Usefulness	Likelihood of implementing the strategy
	Median (Q1–Q3)	Median (Q1–Q3)	Median (Q1–Q3)
Understanding of harm reduction practices	4 (3–5)	5 (5–5)	5 (4–5)
Knowing about stimulant half-life	0 (0–4)	5 (5–5)	4.75 (4–5)
Considering the peak effect of a substance	3 (1–4)	5 (5–5)	4.25 (3.7–5)
Considering high-risk doses	3 (2–5)	3 (2–5)	3 (2–5)
Module 1			
Healthy eating	3 (3–4)	5 (5–5)	5 (4–5)
Sleeping hygiene	3 (2–4)	5 (5–5)	5 (4–5)
Oral care	4 (3–4)	4 (3–4)	4 (3–4)
Skincare	4 (3–5)	5 (5–5)	5 (5–5)
Mental health care	3 (3–4)	5 (5–5)	5 (5–5)
Sexual health care	3 (3–4)	5 (4–5)	5 (4–5)
Considering expenses and making a budget	3 (1–4)	5 (5–5)	5 (4–5)
Considering tips for taking care of your economy	3 (3–4)	5 (5–5)	5 (4–5)
Context assessment	4 (3–5)	5 (5–5)	5 (5–5)
Location sharing	4 (3–4)	5 (5–5)	5 (4–5)
Asking for a photo of the person they will be meeting	4 (3–5)	5 (5–5)	5 (5–5)
Having emergency contact numbers	3 (0–4)	5 (5–5)	5 (4–5)
Module 2			
Knowledgeable of the potency of substances	2 (0–3.25)	5 (5–5)	5 (4–5)
Spaced substance use	2.5 (1–4)	5 (5–5)	5 (3.7–5)
Checking the table of risky substance combinations	3 (0–4)	5 (5–5)	5 (4–5)
Eating and hydration during sessions	3 (2–4)	5 (5–5)	5 (4–5)
Alarms and scheduled eating times	2 (0–3.25)	5 (5–5)	5 (4–5)
Personal monitoring to assess substance use	3 (2.75–4)	5 (5–5)	5 (4–5)
Assessing risks for a safe return home	4 (2.75–5)	5 (5–5)	5 (4.7–5)
Module 3			
Reestablishing activities after using sessions	3 (2–4)	5 (5–5)	5 (4–5)
Action plan	3 (2–3.5)	5 (5–5)	5 (4–5)
Others			
Regular medical checkups	4 (3–5)	5 (4.5–5)	5 (4–5)
Working out	2 (1–4)	5 (5–5)	5 (3–5)
Avoiding unwanted effects	3.5 (2–4.25)	5 (5–5)	5 (3.7–5)
Avoiding riskier polysubstance use	3.5 (2.75–5)	5 (5–5)	4 (4–5)
Eye care	3 (2–4)	5 (5–5)	5 (4–5)
HIV testing	5 (3.5–5)	5 (5–5)	5 (5–5)
Hygienic equipment availability	4 (3.75–5)	5 (5–5)	5 (4.7–5)
Safe return	5 (3.75–5)	5 (5–5)	5 (5–5)
Leaving credit cards at home	3 (1–5)	5 (5–5)	5 (5–5)
Driving after the effects wear off	3.5 (2.25–4.75)	5 (5–5)	4 (4–5)
Blocking cards	3 (0–4)	5 (4–5)	4 (4–5)
Recovery after physical and mental exhaustion	4 (3–5)	5 (5–5)	5 (4–5)
Blocking bank app	2 (0–3)	5 (4–5)	4 (3–5)
Limiting cash	4 (3–5)	5 (5–5)	5 (4–5)

Q1: quartile 1. Q3: quartile 3

With few exceptions, the information was considered very relevant and useful (i.e., median scores and Q1 with a score of 5 = very useful), and feasibility was also very high (i.e., median scores and Q1 with values of 4 and 5). Inspection of quartiles showed higher variability of feasibility than of usefulness. “Considering high-risk doses” showed the lowest score (median = 3) for both usefulness and feasibility.

### Potential behavioral change

With the data gathered from the frequency and perceived usefulness, possible behavioral change was measured by subtracting how frequently participants used each strategy from the likelihood of implementing them. Then, the median was obtained for each strategy (Table 4). From the analysis of potential behavioral change, topics such as self-care, financial health, and pharmacological knowledge (*e.g.*, the half-life of stimulants, polysubstance use, and overdose prevention) are the strategies the participants are more likely to implement and use into their daily lives.

**Table 4.** Possible behavioral change (n= 17)

	Potential behavioral change		
	Median (Q1–Q3)	W	Significance *p*
Introduction			
Understanding of harm reduction practices	1 (0–1.37)	34.5	0.17
Knowing about stimulant half-life	3 (0–5)	7	<**0.01**
Considering the peak effect of a substance	1.7 (0–3)	4	**0.03**
Considering high-risk doses	1.5 (0–2)	50	**0.02**
Module 1			
Healthy eating	1 (0–2)	79	**0.02**
Sleeping hygiene	1 (0–1)	73.5	<**0.01**
Oral care	1 (0–1)	78.5	**0.02**
Skincare	1 (0–2)	49	**0.03**
Mental health care	2 (0–2)	76	<**0.01**
Sexual health care	1 (0–2)	58.5	**0.02**
Considering expenses and making a budget	1 (0–3)	79.5	**0.02**
Considering tips for taking care of your economy	1 (0–2)	81	**0.01**
Context assessment	1 (0–2)	54.5	0.05
Location sharing	1 (0–1)	63	0.06
Asking for a photo of the person they will be meeting	0 (0–1)	36	0.12
Having emergency contact numbers	1.5 (0–4)	71.5	**0.01**
Module 2			
Knowledgeable of the potency of substances	2 (0.75–4.25)	80	**0.02**
Spaced substance use	1 (0.75–3)	78	<** 0.01**
Checking the table of risky substance combinations	1 (1–4)	97	**< 0.01**
Eating and hydration during sessions	1 (0–2.25)	59.5	0.02
Alarms and scheduled eating times	2 (0.75–4.25)	78	<** 0.01**
Personal monitoring to assess substance use	1 (0–2)	70.5	**0.01**
Assessing risks for a safe return home	1 (0–2)	47.5	0.05
Module 3			
Reestablishing activities after using sessions	1 (0.5–2)	66	<** 0.01**
Action plan	1 (1–2)	78	<** 0.01**
Others			
Regular medical checkups	1 (0–1)	36	0.12
Working out	1 (1–2)	114.5	<** 0.01**
Avoiding unwanted effects	0.5 (0–2.25)	53.5	0.07
Avoiding riskier polysubstance use	0 (0–1)	22.5	0.17
Eye care	0 (0–2)	43	0.12
HIV testing	0 (0–0.75)	10.5	0.50
Hygienic equipment availability	0 (0–1)	36	0.12
Safe return	0 (0–1)	29	0.13
Leaving credit cards at home	1 (0–3)	45	<** 0.01**
Driving after the effects wear off	0 (0–1)	26.5	0.25
Blocking cards	1 (0–3)	64	<** 0.01**
Recovery after physical and mental exhaustion	1 (0–1)	52	0.09
Blocking bank app	2 (0–3)	84.5	<** 0.01**
Limiting cash	0 (0–1)	27.5	0.20

W= Wilcoxon test; Significance at *p *< 0.05 bolded; Q1: quartile 1. Q3: quartile 3. The median score is the difference between the likelihood of implementing minus the frequency of use for each strategy

## Discussion

In this study, we described the design and delivery of a harm reduction psychosocial intervention for stimulant misuse in persons who engage in sexualized substance use. We also assessed its feasibility, acceptability, perceived usefulness, and potential behavioral change among a sample of Mexican gbMSM adults who, mostly, were highly educated and living with HIV. This short but comprehensive intervention had high feasibility (84% completion rate) after enrollment. The intervention was perceived to be acceptable to the participants, and the strategies delivered were rated as very useful and highly feasible to implement. We identified several harm reduction strategies with the most potential for change.

As in other settings, both gbMSM and health providers in Mexico reported a lack of interventions for sexualized substance use [29]. Following current recommendations [32, 33], we developed the intervention tailored to the needs, values, beliefs, and behaviors of Mexican gbMSM who engage in sexualized substance use. To this end, we based the contents of the strategies mainly on three sources of information: (1) members of the gbMSM community; (2) health providers who work with persons who use stimulants, including some from harm reduction CBOs; and (3) evidence-based harm reduction recommendations from high-income countries adapted to the Mexican population [29, 30, 35]. Furthermore, the final version of the intervention was also reviewed by a senior pharmacologist researcher and gbMSM who use stimulants. To our knowledge, this is the first Mexican intervention which contents were designed with these characteristics. The high scores for the perceived usefulness of the strategies may reflect the appropriateness of this aspect of the intervention.

Other features related to the delivery of the intervention should be discussed, including place, time, materials/organization, and facilitators. Although we used public settings to deliver the intervention in-person, some participants commented on the benefits of having other modalities, such as online (either synchronous or asynchronous). These modalities have been explored in harm reduction by mailing naloxone and injection equipment and in psychosocial support [56, 57]. Our strategy to let the participants determine when and where they attend was optimal for this in-person intervention; however, based on the low recruitment rate for this study (35%), we need to find methods to enhance the participation of those who were initially interested in receiving this harm reduction program [58]. A possible explanation for this rate is that the research team was not allowed to provide incentives for participants, which are not only widely used, but also recommended or required in high-resource settings in which most of the interventions are designed and implemented. Importantly, the public clinic was the location in which more participants attended the intervention. The clinic is widely recognized as a friendly place for persons at higher risk of or living with HIV. This is one of the strengths of the study, considering that it targeted key populations that are already enrolled in public health care for other reasons than that of stimulant use. As stated before, previous research has mostly targeted persons disenfranchised with the health system. Based on this, it is recommended to strengthen the liaison between academia and practitioners in implementation research [59]. Future studies, including the effectiveness assessment of this intervention, may benefit from continuing collaborating with public clinics.

Based on the low desertion and high perceived usefulness, the materials and organization of how the strategies were delivered were appropriate, and they could be used to scale the reach of the intervention. However, to accomplish this, the facilitators should be extensively trained in substance use and harm reduction strategies, such as those in this study. Overall, the intervention was considered very acceptable by the participants. This included that they considered it clear, useful, and that it may improve their health. Prior studies have analyzed the acceptability of online interventions related to substance use [60–62]. However, to our knowledge, this is the first study that sought to assess the acceptability of an in-person harm reduction intervention.

Overall, participants were somewhat familiar with several of the strategies of the intervention. However, two sets of strategies stood out. First, although participants had a high educational attainment, they had little knowledge on basic psychopharmacological effects of the substances they frequently use. This strategy was especially important for understanding potential risks for overdosing and riskier substance combinations, as sexualized substance use is highly frequent among gbMSM who already use substances [63]. By addressing this topic, the present intervention may reduce riskier polysubstance use. Second, other strategies not frequently used prior to the intervention were related to financial health. However, participants acknowledged the importance of including these strategies. This is an important opportunity to improve the well-being of gbMSM who engage in sexualized substance use considering the heightened risk of being victims of delinquency while under the effects of substances [64].

The information–motivation–behavioral (IMB) Skills Model, which has been widely used in HIV prevention and treatment interventions [65, 66], may also help interpret our findings. The IMB model considers the information, motivation, and behavioral skills that are related to health behaviors [67]. Regarding potential behavioral change, we found that participants reported to be more likely to incorporate information related to pharmacological characteristics of stimulants in their subsequent use. This finding contradicts stigmatizing beliefs on persons who use drugs in terms of their lack of self-care. By providing information on substances half-lives and riskier combinations, persons may be more prepared to plan and select which substances they will use prior to a session. This is similar to other harm reduction techniques such as drug-checking, which has proved to be related to behavior change [68–70].

The design and implementation of this harm reduction intervention had several limitations. First, due to the recruitment strategy, we were unable to determine the characteristics associated with loss of potential participants from the first contact to the intervention date. However, most of the participants who were enrolled completed the intervention, which indicates high feasibility for these participants. Second, although the intervention was based on the needs of gbMSM who engage in sexualized substance use, none of the persons that administered the intervention had lived experience with this substance use. This did not appear to be a major limitation, but it is recommended that future studies include persons with lived experience. Third, although the intervention was designed using the common language of 8th grade, we were not able to assess whether the language was accessible for persons with low educational attainment since our participants were highly educated. Fourth, this study reports on the first phases of implementation research, but more work is needed to assess effectiveness of the strategies in behavior change.

Despite these limitations, this feasible, acceptable, and community evidence-based intervention has the potential to reduce the harm associated to the sexualized substance use of stimulants among gbMSM in low and middle-income settings. Harm reduction interventions need to address gbMSM health risks in a broader manner that includes a wider scope of strategies other than condom use and HIV prevention, testing, and treatment. This may include pharmacological information on common substances and broader self-care strategies. It is especially recommended to implement it in public health services where potential participants are already engaged.

## Data Availability

The datasets used and/or analysed during the current study are available from the corresponding author on reasonable request.
